# Anthraquinones Inhibit Insulin Amyloidosis in Crowded Environments

**DOI:** 10.3390/molecules31071092

**Published:** 2026-03-26

**Authors:** Jiaxing Zhang, Wen Wang, Zubiyan Yibula, Xin Peng, Rongxin Su, Wei Qi

**Affiliations:** 1 School of Life Sciences, Faculty of Medicine, Tianjin University, Tianjin 300072, China; zubiyan_123@tju.edu.cn; 2 Chemical Engineering Research Center, School of Chemical Engineering and Technology, Tianjin University, Tianjin 300072, China; zhangjiaxing7137@tju.edu.cn (J.Z.); 13193991366@163.com (W.W.); surx@tju.edu.cn (R.S.); 3 State Key Laboratory of Chemical Engineering and Low-Carbon Technology, Tianjin University, Tianjin 300072, China; 4 Guangdong Provincial Key Laboratory of Utilization and Conservation of Food and Medicinal Resources in Northern Region, Shaoguan University, Shaoguan 512005, China; 5 Collaborative Innovation Centre of Chemical Science and Engineering (Tianjin), Tianjin 300072, China; 6 Tianjin Key Laboratory of Membrane Science and Desalination Technology, Tianjin University, Tianjin 300072, China

**Keywords:** anthraquinones, insulin, amyloid fibrillation, macromolecular crowded environment, molecular simulations

## Abstract

Natural anthraquinones possess a wide range of biological activities, including antibacterial, antiviral, antitumor, and antioxidant effects. However, studies on their ability to inhibit amyloid protein aggregation remain relatively limited. In this study, we used insulin as a model protein to investigate the anti-amyloidogenic potential of several natural anthraquinones. Specifically, the inhibitory mechanisms of five anthraquinones (emodin, anthraflavin, aloe-emodin, alizarin, and purpurin) on insulin amyloid fibrillation were explored in both dilute and crowded environments (PEG 2000 and PEG 4000). Multidisciplinary analytical results demonstrated that all five anthraquinones could effectively inhibit insulin amyloid fibrillation in both dilute and crowded environments. Simultaneously, crowded agents themselves also exhibited inhibitory effects on insulin amyloid aggregation. However, the inhibitory efficacy of anthraquinones was weaker in crowded environments than in dilute solutions, indicating that although crowded agents themselves suppressed insulin aggregation, they may interfere with the regulatory roles of anthraquinones on insulin aggregation behavior. Interestingly, purpurin showed stronger inhibitory activity in crowded environments compared to dilute solutions. Furthermore, fluorescence spectral analysis suggested that the quenching mechanism of insulin by all these anthraquinones was identified as static quenching mode. Molecular simulation studies revealed that anthraquinones could bind to the aggregation-prone regions of insulin via hydrogen bonding and hydrophobic interactions, thereby inhibiting insulin amyloid aggregation. Notably, the inhibitory capacity of these compounds was correlated with their structural features and the binding affinities to insulin. Collectively, this study explored the anti-amyloid activity of anthraquinones, which held significant research value for the development of potential therapeutic agents for amyloid-associated proteinopathies.

## 1. Introduction

Insulin and human islet amyloid polypeptide (hIAPP), co-secreted by pancreatic β-cells, are both amyloidogenic proteins/peptides. Their misfolding and aggregation into cytotoxic species are central to the pathogenesis of several debilitating diseases, which highlights an urgent need for effective aggregation inhibitors [1,2]. The pathological aggregation of insulin itself, known as insulin-derived amyloidosis, constitutes a significant clinical complication of long-term insulin therapy for diabetes. These amyloid fibrils accumulate at insulin injection sites, leading to local inflammation, reduced therapeutic effectiveness, and potential immune reactions. More importantly, insulin could rapidly coaggregate with other soluble proteins, including bovine serum albumin and lysozyme [3,4]. In some cases, such heterologous interactions triggered amyloid cross-seeding more efficiently than self-seeding. These findings revealed potential interactions between insulin and other amyloidogenic proteins, emphasizing the critical importance of developing inhibitors that could disrupt these cross-seeding events.

Therefore, there is a considerable and urgent effort to design new drugs that can specifically inhibit the amyloid fibrillation of insulin and its associated proteins/peptides. Traditional stabilizers, such as phenol in commercial insulin formulations, have inherent cytotoxic limitations, driving the search for safer, more effective alternatives [5]. Recently, novel therapeutic strategies have been continuously emerging. For instance, the small molecule PAD-S has demonstrated high potency in binding native insulin, significantly delaying its fibril formation and preventing the associated self-assembly process [6]. Other studies have focused on stabilizing the spatial conformation of insulin. Recent studies found that zinc and specific pH conditions can determine dynamical properties and aggregation kinetics of human insulin from forming harmful aggregates, providing a blueprint for engineering more effective therapeutic molecules [7,8]. Ultra-short peptides have also been confirmed to exert inhibitory effects on insulin aggregation [9,10]. Notably, despite their potential photosensitizing activity under specific conditions, anthraquinones exhibited favorable biocompatibility and intrinsic antioxidant properties, enabling them to function as effective suppressors of insulin amyloidogenesis [11,12,13]. In general, the inhibitor molecules could interact with the active region of amyloid peptides, which are prone to aggregation, thereby blocking the process of amyloid fibrosis [14,15]. These research directions not only aimed to improve diabetes therapy by preventing injection-site amyloidosis but also sought to develop agents that could mitigate the broader, interconnected pathological conditions between diabetes and other aggregation-associated diseases [16].

Although extensive studies have been conducted on the inhibition of amyloid aggregation, most of the works were performed in dilute solutions in vitro, which was very different from the actual environment inside the cell. In fact, proteins exerted their biological functions within highly crowded compartments such as the cytoplasm, nucleus, and blood plasma, which were densely packed with diverse macromolecules [17]. Notably, significant differences are often observed when investigating the physiological activities of proteins in dilute solutions versus crowded environments. This was because, compared with dilute solution systems, macromolecular crowders occupied a dominant position in crowded environments, with a high occupied volume fraction. This characteristic (named the macromolecular crowded effect or excluded-volume effect) increased the steric hindrance of the system, limited the activity space of proteins, and ultimately led to alterations in the properties of biomolecules and related biological processes [18,19]. Therefore, the crowded environment was an indispensable factor that must be accounted for in any physiologically relevant investigation of protein function and amyloid formation.

The crowded environment within the organism exerts significant impacts on the thermodynamics, kinetics, and structural properties of biological macromolecules [20]. Proteins usually exist in naturally crowded milieus such as blood or cells. In comparison with the dilute solution systems, the crowded environments can induce specific changes in protein structure and conformation. In the past, our understanding of the interaction mechanisms between small molecules and proteins, as well as the behaviors of protein conformational changes, has been derived from studies conducted in dilute solution systems. However, the experimental results obtained in this way often deviate from the real in vivo situation. Therefore, to more accurately understand the behaviors of proteins within organisms, it has been proposed that appropriate crowded reagents should be added to traditional research systems to simulate the real crowded environment present in living organisms [21]. An ideal crowded environment should closely resemble the real in vivo condition, and the optimal approach is to use cell extracts as crowding reagents. Nevertheless, the heterogeneity of the chemical, geometric and physical properties of cell extracts rendered the collection and analysis of experimental data extremely challenging. Additionally, there was a lack of suitable theoretical models to interpret the experimental findings [22]. As a practical alternative, inert polymers were commonly employed to simulate the crowded phenomenon in controlled settings. Among them, polyethylene glycol (PEG), a water-soluble, electrically neutral and biocompatible polymer, was widely used due to its chemical inertness, low toxicity, and well-characterized behavior in biological contexts. PEG-based crowding systems thus provided a tractable and physiologically informative platform for studying protein behavior under near-native conditions.

**Figure 1:**
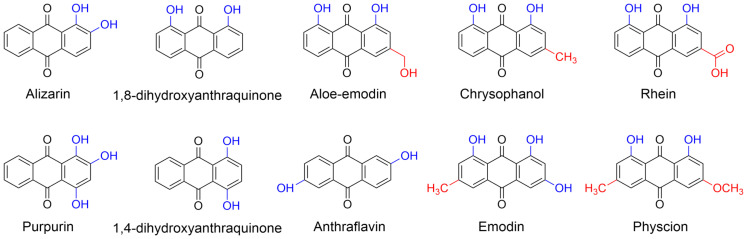
The chemical structures of ten anthraquinones involved in this study.

In this study, we investigated the effects of crowded reagents on the intrinsic fluorescence and structure of insulin by employing PEG 2000 and PEG 4000 to simulate physiologically crowded environments. Steady-state fluorescence spectroscopy was then used to study the interaction mechanisms between insulin and a series of anthraquinones (Figure 1), including alizarin and purpurin described in our previous work [23], in both dilute solutions and crowded environments. Subsequently, the insulin fibrilization and the corresponding inhibition effects of various anthraquinones in the absence and presence of crowding agents were analyzed by Thioflavin-T (ThT) fluorescence spectroscopy, circular dichroism (CD), atomic force microscopy (AFM) and dynamic light scattering (DLS). Finally, molecular simulation methods were performed to elucidate the molecular mechanisms underlying anthraquinone-mediated inhibition of insulin aggregation. In short, this work provided a comprehensive assessment of how natural anthraquinones suppressed insulin amyloid formation in a simulated crowded environment, offering valuable insights for the rational design of therapeutic agents targeting protein misfolding disorders.

## 2. Results and Discussion

### 2.1. Characterization of the Inhibitory Effects of Anthraquinones on Insulin Fibrillation

Based on a previous study in our laboratory [23], we selected ten structurally similar anthraquinones featuring varying numbers and positions of hydroxyl and methyl groups (Figure 1) to study their inhibitory effects on insulin amyloid fibrillation. All these anthraquinones reduced the ThT fluorescence intensity of insulin aggregates, where emodin, anthraflavin and aloe-emodin showed the best inhibition performance, thereby demonstrating the greatest potential for further investigation (Figure S1). Hence, alizarin, purpurin, emodin, anthraflavin and aloe-emodin were chosen as representative anthraquinones for detailed studies on their inhibitory activities of insulin aggregation in crowded environments.

To screen an appropriate crowded reagent, we investigated the effects of five commonly used crowded reagents (PEG 2000, PEG 4000, PEG 6000, PEG 8000 and PEG 10000) on the intrinsic fluorescence intensity of insulin (Figure S2). All these PEGs induced a concentration-dependent decrease in insulin’s fluorescence intensity, albeit with different quenching degrees. Among them, PEG 2000 and PEG 4000 exhibited the lowest quenching rate constants toward insulin (Table S1), which were much lower than the maximum diffusion–collision quenching rate constant (2 × 10^10^ M^−1^·s^−1^), suggesting that the quenching mechanism was dynamic quenching rather than static quenching (ground-state complex formation), which meant that these two PEGs exerted minimal effects on insulin conformations. Thus, PEG 2000 and PEG 4000 were prepared at a common concentration of 100 g/L to mimic the in vitro crowded environment, which was used to study the inhibitory effect of anthraquinones on insulin amyloid fibrillation in a macromolecular crowded environment.

**Figure 2:**
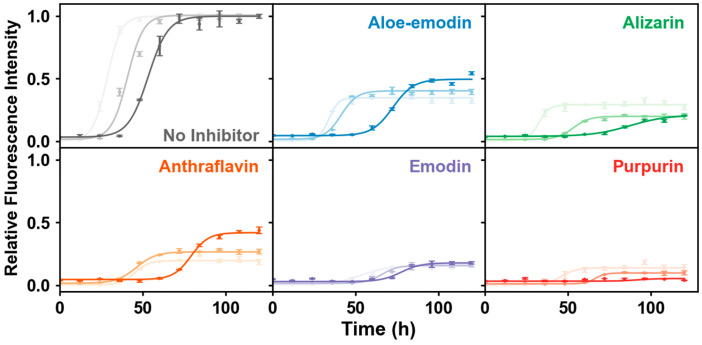
Time-dependent normalized ThT fluorescence curves for insulin aggregation without and with five distinct inhibitors in the presence of different crowded reagents. The color brightness of curves corresponds to different experimental environments: light color (dilute solution), medium color (PEG 2000), and dark color (PEG 4000).

**Figure 3:**
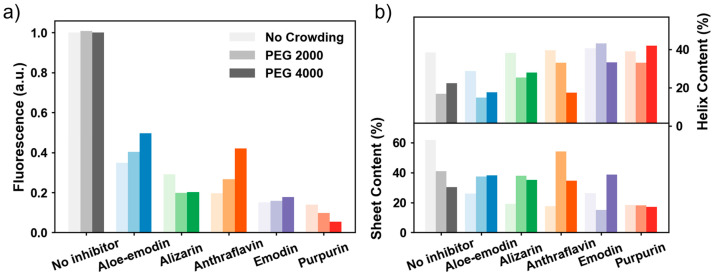
The aggregation-inhibition efficacy of anthraquinones on insulin. (**a**) Normalized ThT fluorescence intensity at the plateau stage. (**b**) α-helix and β-sheet structure contents of insulin samples after 84 h of incubation in different crowded environments. The color brightness of curves corresponds to different experimental environments: light color (dilute solution), medium color (PEG 2000), and dark color (PEG 4000).

To measure the effects of anthraquinones on insulin fibrillation in crowded environments, we used the amyloid-specific fluorescent probe ThT [24] to monitor the amyloid aggregation process. In the absence of anthraquinones, the ThT fluorescence intensity of insulin decreased with the increasing crowded extent of the system (Figure S3). This observation reflected that the crowded reagent itself exerted an inhibitory effect on insulin aggregation. Thus, we used relative fluorescence intensity to assess the inhibition ability of anthraquinones on the kinetics of insulin aggregation in both dilute solutions and crowded environments (Figure 2 and Figure 3a, Table S2). For pure insulin, the ThT fluorescence intensity gradually increased with incubation time and finally reached a plateau. In contrast, in the presence of anthraquinones, insulin aggregates showed decreased ThT fluorescence intensity and an extended nucleation period, regardless of whether the system was a dilute solution or a crowded environment supplemented with PEG 2000/PEG 4000. This result demonstrated that all anthraquinones displayed inhibition effects on insulin aggregation. Among these compounds, purpurin and emodin showed the most significant inhibition activities, followed by the other three anthraquinones. Notably, anthraflavin showed stronger inhibition ability in dilute solutions than in crowded environments. In addition, the inhibitory effects of most anthraquinones were weaker in crowded environments compared to dilute solutions. This phenomenon may be attributed to the excluded-volume effect or an increase in the system viscosity, which enhanced the resistance of inhibitors to contact with insulin. This hindrance prevented their mutual binding and thereby weakened the inhibitory effect, which was consistent with the changing trend of the binding constant described in the following section.

### 2.2. Analysis of Interaction Mechanisms Between Insulin and Anthraquinones

It is well established that proteins contain aromatic amino acid residues, such as tyrosine, phenylalanine and tryptophan, that can exhibit intrinsic fluorescence. Upon binding to a ligand, this inherent fluorescence is often quenched due to changes in the local microenvironment or direct interactions with the fluorophores [25]. This property makes intrinsic fluorescence a powerful tool for probing protein–ligand interactions. Accordingly, the fluorescence spectral method was utilized to investigate the interaction mechanisms between the anthraquinones and insulin. It could be observed that in these three research systems, as the concentration of inhibitors increased, the fluorescence intensity of insulin gradually decreased, indicating that interactions had occurred between these anthraquinones and insulin (Table 1, Figures S4 and S5). Using the Stern–Volmer equation, the *K*_q_ values of all anthraquinone–insulin systems could be obtained. Obviously, for all tested anthraquinones, the calculated *K*_q_ values were higher than the maximum diffusion-collision quenching rate constant (2 × 10^10^ M^−1^·s^−1^), indicating that the quenching process of insulin by anthraquinones was a static quenching mechanism, i.e., the formation of stable non-fluorescent insulin–anthraquinone complexes. Moreover, the *n* values were close to 1 for all anthraquinones regardless of the presence of crowding reagents, indicating the presence of a single primary binding site on insulin. In addition, purpurin and emodin showed the highest binding constants, suggesting their outstanding binding affinity toward insulin. This superior binding capacity may be attributed to the fact that purpurin and emodin possessed more hydroxyl groups than the other anthraquinones, which in turn facilitated stronger hydrogen bonding with insulin.

### 2.3. Circular Dichroism Study

CD spectroscopy has been widely used to analyze the secondary structure changes of amyloid proteins during the fibrosis process (Figure 3b) [26]. The CD spectra of native insulin in all three tested environments showed typical α-helical absorption peaks at 208 nm and 220 nm (Figure S6). This observation indicated that the crowding reagents did not affect the native conformation of insulin, thereby confirming that PEG 2000 and PEG 4000 were suitable for use as crowded reagents in in vitro experiments. In the absence of anthraquinones, a prominent negative absorption peak at 218 nm appeared after 84 h of incubation in the three environments, which was indicative of insulin’s conversion into mature amyloid fibrils featuring a typical β-sheet structure. When 200 μM anthraquinones were present in these three environments, the signal peaks at 218 nm corresponding to β-sheet structures were significantly weakened. In the dilute solution containing purpurin and emodin, insulin exhibited the weakest signal peak at 218 nm, demonstrating that purpurin and emodin exerted the best protection effects among the five anthraquinones tested. In the crowded environment, although the inhibitory effect of anthraquinones was somewhat reduced due to increased viscosity or steric constraints caused by crowded reagents, their capacity to lower β-sheet content remained evident compared to untreated insulin aggregates. In particular, in the PEG 4000-induced crowded environment, the CD spectrum of insulin treated with purpurin was very similar to that of natural insulin. Consequently, it could be inferred that purpurin was capable of completely inhibiting insulin amyloid fibrosis in the presence of PEG 4000. Overall, all five anthraquinones maintained a protective or stabilizing effect on the native structure of insulin in both dilute solutions and crowded environments. These effects were approximately consistent with the order of binding constants (Table 1) and the inhibition rates of amyloid aggregation (Figure 2).

### 2.4. Atomic Force Microscopy Study

**Figure 4:**
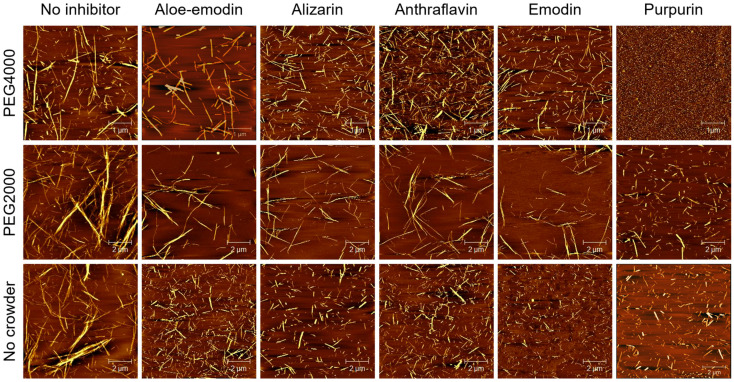
AFM images of insulin amyloid fibrillation without and with five anthraquinones in different crowded environments.

The morphology of insulin aggregates in the presence of anthraquinones was explored by AFM (Figure 4). It was clear that anthraquinones reduced the density or size of insulin aggregates and suppressed the amyloid fibrosis of insulin when compared with pure insulin. In dilute solutions, insulin fibers treated with anthraquinones obviously showed a marked reduction in density and a shortening in length. In the PEG 2000-induced crowded environment, the morphology of insulin aggregates treated with anthraquinones also showed a decrease in density. However, the reduction in fiber length was less pronounced than that observed in the dilute solution, indicating that the crowded environment could modulate the inhibitory effect of anthraquinones. For PEG 4000, the morphology of pure insulin aggregates was shorter than that in the other two research systems, and the length of insulin fibers was also reduced, which was consistent with the previous ThT fluorescence spectroscopic analysis. In addition, purpurin-treated insulin samples were found to exhibit a uniform punctate morphology, which was highly similar to the morphological features of native insulin, indicating that purpurin could completely inhibit the amyloid fibrosis of insulin after adding PEG 4000. Overall, all anthraquinones exerted inhibitory effects on the insulin amyloid fibrosis process in both dilute solutions and crowded environments. Among these compounds, emodin and purpurin exhibited the strongest inhibitory effect, which almost completely suppressed the aggregation behavior of insulin in the PEG 4000-induced crowded environment.

### 2.5. Dynamic Light Scattering Study

**Figure 5:**
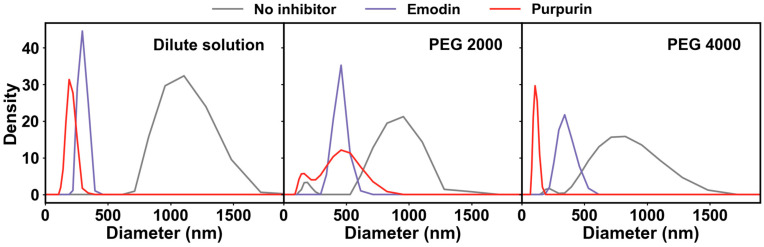
Size distribution of the insulin aggregates incubated for 84 h without and with emodin and purpurin in dilute solutions and crowded environments.

To further investigate the effects of emodin and purpurin on the size of insulin aggregates, DLS analysis was performed in both dilute and crowded environments (Figure 5, Table S4). Upon co-incubation with either emodin or purpurin, the particle size of insulin in all three tested environments was significantly reduced, indicating that the introduction of emodin and purpurin could suppress insulin amyloid fibrosis and diminish the size of insulin aggregates. For the same anthraquinone, its inhibitory effect on insulin aggregates weakened as the crowded degree of environment increased. Notably, in the PEG 4000-induced crowded environment, purpurin was able to keep the particle size of insulin at a level close to that of native insulin (~100 nm), indicating that the amyloid fibrosis process of insulin was fully inhibited under this condition, which was consistent with the results of previous experimental studies.

### 2.6. Molecular Interactions Study

**Figure 6:**
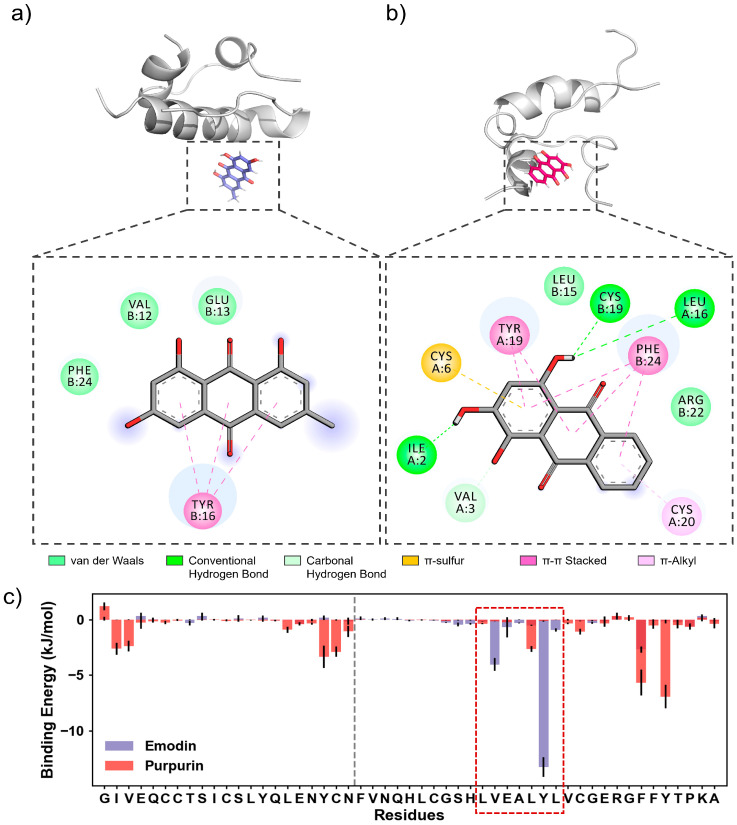
Molecular structures of (**a**) the insulin–emodin complex and (**b**) the insulin–purpurin complex after 100 ns MD simulations and the corresponding interactions shown in a 2D format. (**c**) Binding energy decomposition on insulin residues during MD simulations.

To further explore the interaction mechanisms between anthraquinones and insulin, molecular docking and molecular dynamics simulation (MD) studies were carried out on the insulin–emodin system and insulin–purpurin system. After molecular docking and 100 ns MD simulations of these two complexes (Figure 6a), it was found that purpurin could insert into the interface between the A and B chains of insulin, whereas emodin interacted with the B11–B17 hydrophobic core (^11^LVEALVL^17^) of the B chain (Figure 6b) [27]. The decomposition of binding energy also confirmed that emodin showed significant interactions with the B11–B17 hydrophobic core (red box in Figure 6c), while purpurin interacted with multiple residues in both chains of insulin. As a result, purpurin displayed a more excellent anti-aggregation effect on insulin aggregation. During the stable stage of MD simulations (60–80 ns), the purpurin molecule showed stronger interactions with insulin than emodin (Table 2), with a binding free energy difference of approximately 34 kJ/mol. Additionally, the counts of α-helix and β-sheet residues within the two simulated systems also revealed that purpurin had an effect of reducing the β-sheet structure (Figure S7). In a word, the molecular simulation results theoretically verified that both emodin and purpurin showed effective inhibitory activity against insulin aggregation, among which purpurin was more potent.

### 2.7. Pharmacokinetic Property Analysis

To further evaluate the pharmacokinetic properties of anthraquinones including emodin and purpurin, we performed in silico predictions of their lipophilicity (Table S5). Lipophilicity, commonly reflected by partition coefficient Log P, serves as a pivotal factor dominating drug membrane permeability, bioavailability, and tissue distribution. The consensus Log P values were determined to be 1.87 for emodin and 1.88 for purpurin, close to the optimal range of 2–4 for drug-like small molecules, suggesting favorable intestinal absorption capacity. In line with this result, both compounds were predicted to possess high gastrointestinal absorption and fully comply with Lipinski’s rule of five. These findings align with the reported pharmacokinetic behaviors of free anthraquinones, which exhibit rapid intestinal absorption and widespread tissue distribution due to their moderate lipophilicity [28]. Overall, the desirable lipophilicity characteristics of emodin and purpurin further support their application potential as therapeutic candidates for amyloid-associated disorders.

## 3. Materials and Methods

### 3.1. Materials

Bovine insulin was obtained from Absin Bioscience Inc. (Shanghai, China). The anthraquinones, including emodin, rhein, aloe-emodin, chrysophanol, anthraflavin, 1,4-dihydroxyanthraquinone, alizarin and purpurin, were purchased from Shanghai Aladdin Biochemical Technology Co., Ltd. (Shanghai, China). 1,8-Dihydroxyanthraquinone and physcion were sourced from Sigma-Aldrich (St. Louis, MO, USA) and Beijing J&K Scientific Co., Ltd. (Beijing, China), respectively. Polyethylene glycols (PEGs) of various molecular weights were used as macromolecular crowded agents to simulate cellular environments. PEG 2000, PEG 4000, PEG 6000, and PEG 8000 were acquired from Tianjin Guangfu Fine Chemical Research Institute (Tianjin, China), while PEG 10000 was obtained from Tianjin Tiantai Fine Chemicals Co., Ltd. (Tianjin, China). Thioflavin T (ThT), a fluorescent dye for amyloid fibril detection, and sodium chloride (NaCl) were purchased from Sigma-Aldrich (St. Louis, MO, USA). Acetic acid (HAc) was obtained from Tianjin Concord Technology Co., Ltd. (Tianjin, China). All reagents were of analytical grade and used without further purification.

### 3.2. Solution Preparation

Preparation of Anthraquinone Solutions: For the ten anthraquinones studied in the experiments, an appropriate amount of each anthraquinone was accurately weighed using an electronic balance and dissolved in absolute ethanol. After full dissolution, 2 mM stock solution was obtained and stored at 4 °C.

Preparation of Insulin Solution: Lyophilized insulin powder was dissolved in 20% (*v*/*v*) acetic acid (pH = 1.9, 50 mM NaCl) and allowed to stand for 15 min in a 4 °C refrigerator to fully dissolve, resulting in an insulin stock solution with a concentration of 1.11 mg/mL.

Preparation of PEG 2000/PEG 4000/PEG 6000/PEG 8000/PEG 10000 Solutions: An accurate mass of 25 g PEG 2000, PEG 4000, PEG 6000, PEG 8000 or PEG 10000 was weighed separately using an electronic balance. Each PEG sample was dissolved in ultrapure water and then diluted to the marked volume in a 50 mL volumetric flask to obtain a 500 g/L stock solution. All stock solutions were stored at room temperature for subsequent use.

### 3.3. Effects of Different Macromolecular Crowded Reagents on the Fluorescence Spectra of Insulin

A fixed volume of insulin solution and varying volumes of macromolecular crowded reagent were added into tubes, yielding a final insulin concentration of 10 μM and crowded reagent concentrations of 0, 25, 50, 75, 100, 125, 150, 175, and 200 g/L. The mixtures were incubated in a constant-temperature water bath at 25 °C with shaking for 30 min to ensure adequate mixing between insulin and the crowded reagent. The intrinsic fluorescence spectra of insulin were then measured using a Cary Eclipse fluorescence spectrophotometer (Varian Inc., Palo Alto, CA, USA). The instrumental parameters were set as follows: excitation wavelength of 280 nm, emission scanning range of 285–350 nm, emission wavelength of 303 nm, excitation and emission slit widths of 10 nm, with detection performed at room temperature under appropriate photomultiplier tube voltage. For each sample, the background signal contributed by the crowded reagent alone at the corresponding concentration was subtracted to eliminate interference.

### 3.4. Effects of Anthraquinones on the Fluorescence Spectra of Insulin in Crowded Environments

Insulin solutions were prepared in the absence or presence of PEG 2000 or PEG 4000 as crowded reagents, with a final insulin concentration of 10 μM and a final crowded reagent concentration of 100 g/L. The mixtures were incubated in a 25 °C water bath for 30 min to ensure thorough mixing. A 2 mM stock solution of anthraquinones was diluted 10-fold with PBS buffer to yield a 200 μM working solution for subsequent fluorescence titration experiments.

Fluorescence Titration Experiment: A 2.0 mL volume of the above insulin-crowded reagent mixture was transferred into a 1.0 cm quartz cuvette. Then, sequential titrations were performed by adding 20 μL of anthraquinone solution (200 μM). After each addition, the mixture was equilibrated for 5 min at 25 °C. Fluorescence spectra were recorded from 285 to 350 nm at an excitation wavelength of 280 nm, with excitation and emission slit widths both set to 10 nm. Background spectra were recorded by titrating 20 μL of the anthraquinone solution into 2.0 mL of 100 g/L crowded reagent solution (in the absence of insulin), and these background signals were subtracted from the corresponding sample spectra.

### 3.5. Preparation of Insulin Amyloid Fibrils

Three different incubation conditions were employed to prepare three experimental sample sets. Group 1: Anthraquinones were added directly to insulin solutions, yielding a final insulin concentration of 1 mg/mL (~175 µM) and a final anthraquinone concentration of 200 µM. Group 2: PEG 2000 was added to the corresponding samples to reach a final concentration of 100 g/L, with the concentrations of insulin and anthraquinone maintained identical to those in Group 1. Group 3: PEG 4000 was introduced to the Group 1 formulation to a final concentration of 100 g/L, while keeping identical concentrations of insulin and anthraquinone.

For the fibrillation assay, 900 µL of insulin solution was added to each incubation tube, followed by 100 µL of anthraquinones at varying concentrations. The final concentration of insulin in the mixture was 1 mg/mL, and the concentrations of anthraquinones were 25, 50, 100 and 200 µM. The tubes were then incubated in a thermostatic water bath at 60 °C with a shaking speed of 25 rpm for a designated duration to induce insulin fibrillation. Samples were collected and analyzed at 12 h intervals.

### 3.6. ThT Fluorescence Spectrum Assay

At designated time points during incubation, 20 µL insulin samples were added to quartz cuvettes containing 2 mL of ThT solution. ThT fluorescence intensity was measured using a Cary Eclipse fluorescence spectrophotometer under the following conditions: excitation wavelength of 440 nm, emission wavelength of 480 nm, scanning range of 450–550 nm, slit widths of 5 nm for both excitation and emission. To eliminate the background signal, control measurements were carried out using identical solutions without insulin, and these background values were subtracted from the corresponding readings of insulin samples. All fluorescence data were acquired in triplicate to ensure reliability.

The insulin amyloid fibrillation process followed a typical sigmoidal kinetic pattern. Accordingly, the ThT fluorescence intensity data obtained at different incubation times were subjected to curve fitting to generate the insulin aggregation kinetic curves and related parameters. The specific form of the logistic equation used is detailed below:

(1) $$\;F=F_{max}+\frac{F_{min}-F_{max}}{1+e^{(t-t_{1/2})/\tau }}\;$$

(2) $$t_{lag}=t_{1/2}-2\tau$$

where *F* is the ThT fluorescence intensity of insulin sample at incubation time *t*, *F*_min_ is the initial fluorescence intensity, *F*_max_ is the maximum fluorescence intensity, *t*_1/2_ is the incubation time at which the fluorescence intensity reaches half of the maximum value, 1/*τ* represents the apparent fibrillation rate constant, and *t*_lag_ is the lag time for insulin aggregation.

### 3.7. Fluorescence Spectroscopy

The fluorescence spectra of insulin were determined using a Cary Eclipse fluorescence spectrophotometer at 25 °C. A 2 mL volume of insulin solution (a final concentration of 10 μM) was transferred into a 1 cm quartz cuvette, followed by the gradual addition of anthraquinone solution (final concentration ranging from 0 to 20 μM). The excitation wavelength was set at 280 nm, and the emission spectrum was scanned over a wavelength range of 290–350 nm, with both excitation and emission slits set to 10 nm. To eliminate background interference, the fluorescence data of equivalent blank solutions (without insulin) were subtracted from the corresponding sample measurements. In addition, all the fluorescence data was corrected to eliminate the influence of the inner filter effect based on the method reported by Peng et al. [29].

To characterize the quenching mechanism, the fluorescence data were analyzed according to the following Stern–Volmer equation:

(3) $$F_{0}/F=1+K_{sv}[Q]=1+K_{q}\tau_{0}[Q]$$

where *F*_0_ and *F* represent the fluorescence intensities of insulin in the absence and presence of anthraquinones, respectively. [Q] refers to the concentration of anthraquinones. *K*_sv_, *K*_q_ and *τ*_0_ denote the Stern–Volmer quenching constant, the biomolecular quenching rate constant and the fluorescence lifetime of the biomolecule without a quencher, respectively.

Typically, under the assumption that small molecules bind independently to a set of equivalent binding sites on a protein, the binding constant (*K*_a_) and the number of binding sites (*n*) could be calculated using the following equation [30]:

(4) $$\;\log\frac{F_{0}-F}{F}=n\log K_{a}-n\log\left[\frac{1}{\left[Q_{T}\right]-\frac{\left(F_{0}-F\right)\left[P_{T}\right]}{F_{0}}}\right]\;$$

where *F*_0_ and *F* are the fluorescence intensities of insulin with and without anthraquinones, and [*Q*_T_] and [*P*_T_] are the total concentrations of anthraquinones and insulin, respectively.

### 3.8. CD Spectroscopy

The secondary structure content of insulin in the three sample groups described in Section 3.4 was determined using a JASCO J-810 circular dichroism spectrometer (Jasco, Hachioji, Japan). CD spectra were recorded under the following conditions: scanning range of 190–260 nm, scanning speed of 100 nm/min, bandwidth of 2 nm and path length of 0.1 mm. A blank solution (without insulin) was employed to acquire the baseline spectrum, which was then subtracted from all CD spectra to obtain the true sample spectra. Each sample was scanned in triplicate, and the average spectrum was obtained. Finally, the secondary structure content of insulin was calculated using the SELCON3 program in the CDPro software (https://www.bmb.colostate.edu/cdpro/, accessed on 22 March 2026).

### 3.9. AFM Measurement

The morphology and structural features of insulin amyloid aggregates were analyzed using the AFM method. The incubated insulin samples were diluted 25-fold with ultrapure water and then deposited onto a freshly cleaved mica sheet. Following a 30 min incubation at room temperature, the mica sheet was gently rinsed three times with ultrapure water to remove residual NaCl and loosely bound aggregates. The morphology of insulin aggregates was observed in air using a Dimension Icon atomic force microscope (Bruker Ltd., Karlsruhe, Germany) under standard scanning mode. The acquired AFM images were processed and analyzed using Gwyddion 2.58 analysis software.

### 3.10. DLS Measurement

The hydrodynamic diameter of insulin samples was determined using the DLS method. Measurements were performed using a Zetasizer Nano-ZS instrument (Malvern Instruments Ltd., Malvern, UK) equipped with a He-Ne laser (λ = 633 nm) operating at a scattering angle of 173°. All analyses were carried out at 25 °C, and the reported hydrodynamic diameter of each sample represented the average of three independent measurements.

### 3.11. Molecular Docking and Molecular Dynamics Simulations

The molecular structure of insulin monomers was acquired from the Protein Data Bank (PDB IDs: 1ZNI). The binding of emodin/purpurin to insulin was investigated by using molecular docking with DSDP software (updated on 27 June 2025) [31]. The docking box was set to cover the entire protein molecule, allowing the two natural anthraquinones to dock onto the surface of insulin. MD simulations of the obtained insulin–emodin system and insulin–purpurin system were then performed using Gromacs 2025.2 software [32] with an Amber ff99SB force field [33]. Both systems were placed within a periodic boundary condition box with a 0.8 nm margin, which was filled with TIP3P water molecules [34]. Thereafter, 0.15 M Na^+^ and Cl^−^ ions were added to maintain the electrical neutrality of the systems. Subsequently, the system was subjected to 2000 steps of energy minimization using the steepest descent method. Finally, a 100 ns simulation was conducted for each system under the isothermal–isobaric (NPT) ensemble at 298.15 K with a time step of 2 fs. The MD trajectories were analyzed using Visual Molecular Dynamics (VMD) software v1.9.3 [35] and Gromacs built-in tools. Additionally, the binding energy was calculated via the s_mmpbsa program 0.9.6 [36].

### 3.12. Pharmacokinetic Property Prediction

We performed in silico lipophilicity predictions of the anthraquinones using the SwissADME online tool (http://www.swissadme.ch/), a widely used platform for ADME profiling [37]. The molecular structures of the anthraquinones (Figure 1) were input as canonical SMILES strings. Lipophilicity was estimated using the consensus Log P value, calculated as the arithmetic mean of five independent predictive models (iLOGP, XLOGP3, WLOGP, MLOGP, and SILICOS-IT), to ensure robustness and accuracy. Additional parameters including gastrointestinal absorption and compliance with Lipinski’s rule of five were also evaluated.

## 4. Conclusions

In this study, the inhibitory effects of anthraquinones on insulin amyloid fibrosis in simulated in vitro crowded environments were investigated. Among the ten different anthraquinones tested, emodin, anthraflavin, aloe-emodin, alizarin and purpurin exhibited significant inhibitory effects on insulin amyloid fibrosis. After investigating various crowded environments, PEG 2000 and PEG 4000 were selected as crowded reagents at a concentration of 100 g/L to establish simulated in vitro crowded environments. Subsequently, the intrinsic fluorescence spectra of five anthraquinones were measured in dilute solutions and PEG 2000/PEG 4000-induced environments, revealing a static quenching mechanism with a single binding site between each anthraquinone and insulin. The binding capacity of emodin and purpurin to insulin was significantly higher than that of the other three anthraquinones. Multiple biophysical experiments demonstrated that these five anthraquinones could also inhibit insulin aggregation in crowded environments, with emodin and purpurin showing better inhibitory effects than the other three compounds. Molecular simulation results suggested that anthraquinones could bind to the hydrophobic core of the insulin B chain through hydrogen bonding and hydrophobic interactions, thereby suppressing insulin amyloid aggregation. In addition, while the crowded reagents themselves could moderately inhibit insulin amyloid aggregation, they generally attenuated the inhibitory potency of most anthraquinones (except for purpurin) on the insulin aggregation process, rendering their effects less effective than those observed in dilute solutions.

Notably, although anthraquinones served as potent inhibitors of protein aggregation, their potential cytotoxicity toward normal cells under specific conditions warranted careful evaluation before therapeutic application. With regard to emodin and purpurin, existing evidence suggested a favorable safety profile within relevant concentrations. For instance, Zhao et al. [38] reported that purpurin showed negligible cytotoxicity on normal primary hepatocytes and WRL-68 cells even at concentrations up to 100 μM, with mechanism studies revealing activation of endogenous antioxidant pathways. Similarly, treatment with emodin at 0.5 μM was shown to upregulate the expression of protective antioxidant enzymes in human embryonic kidney cells (HEK 293) [39]. Collectively, these findings indicated that anthraquinones such as emodin and purpurin can present minimal cytotoxicity within the concentration ranges utilized in this study, thereby reinforcing their biosafety and supporting their potential utility as therapeutic agents targeting amyloid-related pathologies.

## Figures and Tables

**Table 1:** The Stern–Volmer quenching constant (*K_sv_*), bimolecular quenching rate constant (*K_q_*), binding constant (*K_a_*) and the number of binding sites (*n*) of the interaction between insulin and five anthraquinones in three research environments.

Systems	Inhibitors	*K_sv_* (10^4^ M^−1^)	*K_q_* (10^12^ M^−1^ s^−1^)	*K_a_* (10^4^ M^−1^)	*n*
No crowder	Aloe-emodin	1.19 ± 0.04	1.19 ± 0.04	1.57 ± 0.10	0.73 ± 0.03
	Alizarin	2.72 ± 0.03	2.72 ± 0.03	3.02 ± 0.09	0.86 ± 0.04
	Anthraflavin	3.18 ± 0.03	3.18 ± 0.03	3.54 ± 0.08	0.74 ± 0.07
	Emodin	3.34 ± 0.10	3.34 ± 0.10	4.84 ± 0.34	0.77 ± 0.04
	Purpurin	5.12 ± 0.05	5.12 ± 0.05	6.88 ± 0.21	0.76 ± 0.03
PEG 2000	Aloe-emodin	1.07 ± 0.04	1.07 ± 0.04	2.56 ± 0.07	1.27 ± 0.04
	Alizarin	2.57 ± 0.04	2.57 ± 0.04	3.33 ± 0.08	0.98 ± 0.04
	Anthraflavin	2.36 ± 0.05	2.36 ± 0.05	3.22 ± 0.08	1.20 ± 0.05
	Emodin	2.95 ± 0.04	2.95 ± 0.04	4.15 ± 0.11	0.92 ± 0.02
	Purpurin	3.28 ± 0.05	3.28 ± 0.05	4.33 ± 0.13	0.86 ± 0.02
PEG 4000	Aloe-emodin	0.85 ± 0.02	0.85 ± 0.02	1.27 ± 0.04	1.17 ± 0.05
	Alizarin	2.42 ± 0.02	2.42 ± 0.02	2.98 ± 0.12	1.01 ± 0.04
	Anthraflavin	2.21 ± 0.02	2.21 ± 0.02	2.75 ± 0.10	0.93 ± 0.02
	Emodin	2.73 ± 0.02	2.73 ± 0.02	3.92 ± 0.11	1.05 ± 0.04
	Purpurin	2.89 ± 0.03	2.89 ± 0.03	3.88 ± 0.14	0.94 ± 0.03

**Table 2:** Binding energy of emodin and purpurin with insulin, analyzed through MM-PBSA method (in kJ/mol).

	ΔE_bind_	ΔE_interaction_	ΔE_sol-polar_	ΔE_sol-nonpolar_	ΔG
Emodin	−37.607	−95.022	68.156	−10.741	−1.474
Purpurin	−79.153	−146.900	83.268	−15.521	−35.912

## Data Availability

The data presented in this study are available in the article and Supplementary Materials.

## Supplementary Materials

The following supporting information can be downloaded at: https://www.mdpi.com/article/10.3390/molecules31071092/s1. Figure S1: Insulin aggregation kinetics curve (A and C) and the final fluorescence intensity (B and D) in the presence of eight anthraquinone compounds at concentrations of 100 μM and 200 μM, respectively; Figure S2: The effect of PEG 2000 (A), PEG 4000 (B), PEG 6000 (C), PEG 8000 (D) and PEG 10,000 (E) on the fluorescence spectrum of insulin. Stern–Volmer curves (F) of insulin incubated with five crowding reagents; Figure S3: The aggregation kinetic curve of insulin samples without inhibitors (A) and the ThT fluorescence spectrum of insulin samples after incubating 84 h (B) in three research environments; Figure S4: Fluorescence spectra of the interaction of aloe-emodin, alizarin, anthraflavin, emodin, and purpurin with insulin; Figure S5: Stern–Volmer (A–C) and modified Scatchard (D–F) curves plots of the interaction of insulin with five anthraquinone compounds in three research environments; Figure S6: The CD spectra of insulin incubated with five anthraquinone compounds in (A) dilute solution, (B) PEG 2000 and (C) PEG 4000 crowding environment for 84 h; Figure S7: The secondary structure residue counts of insulin simulated with emodin and purpurin after 100 ns MD simulations; Table S1: The Stern–Volmer quenching constant (*K*_sv_) and bimolecular quenching rate constant (*K*_q_) of the interaction between insulin and PEG 2000/PEG 4000/PEG 6000/PEG 8000/PEG 10,000; Table S2: Effects of five anthraquinone compounds on the kinetic parameters of insulin aggregation in three research environments; Table S3: Secondary structural contents of insulin in the absence and presence of five anthraquinones after 84 h of incubation; Table S4: Sizes of the insulin–emodin/purpurin in three research environments. Table S5: Basic information and in silico lipophilicity parameters prediction.
