# Supplementary material for: Anthraquinones Inhibit Insulin Amyloidosis in Crowded Environments
Source: Molecules. 2026 Mar 26;31(7):1092. doi: 10.3390/molecules31071092 (PMC13075106; doi:10.3390/molecules31071092)
Supplement: Supplementary file 1 [file molecules-31-01092-s001.zip › molecules-4191552-supplementary.pdf]

Supporting information

Figures

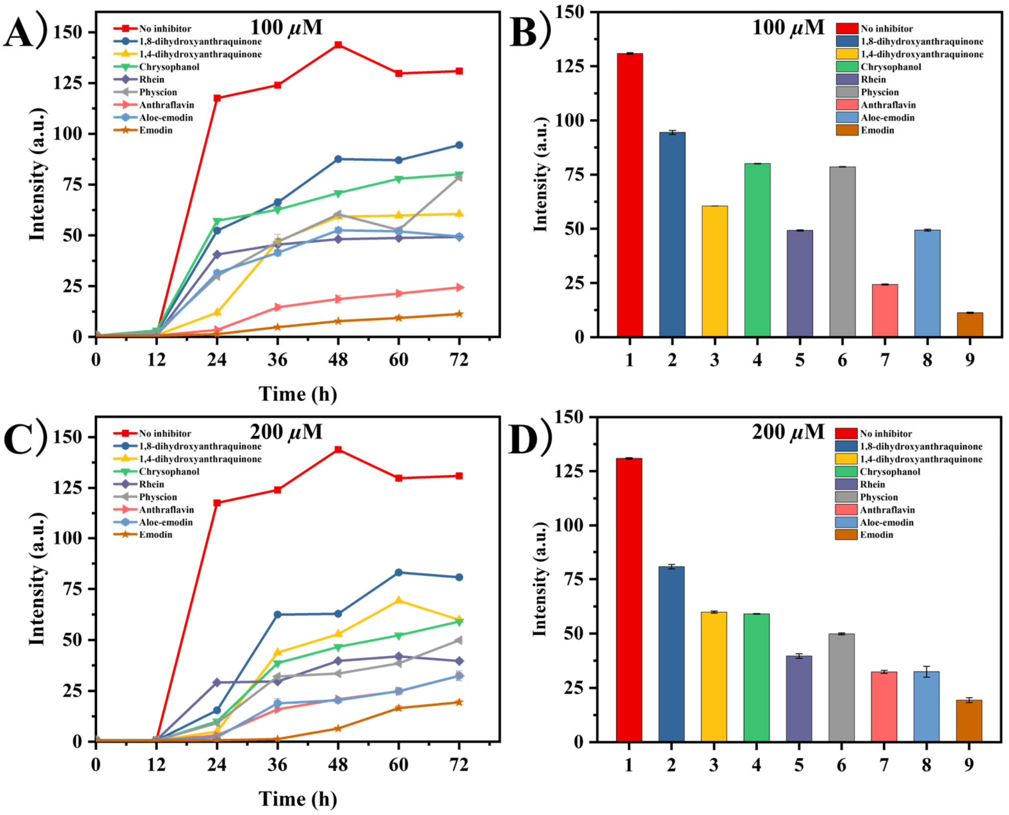

Figure S1. Insulin aggregation kinetics curves (A and C) and the corresponding fluorescence intensity at the plateau stage (B and D) in the presence of eight anthraquinones at concentrations of 100 μM and 200 μM, respectively.

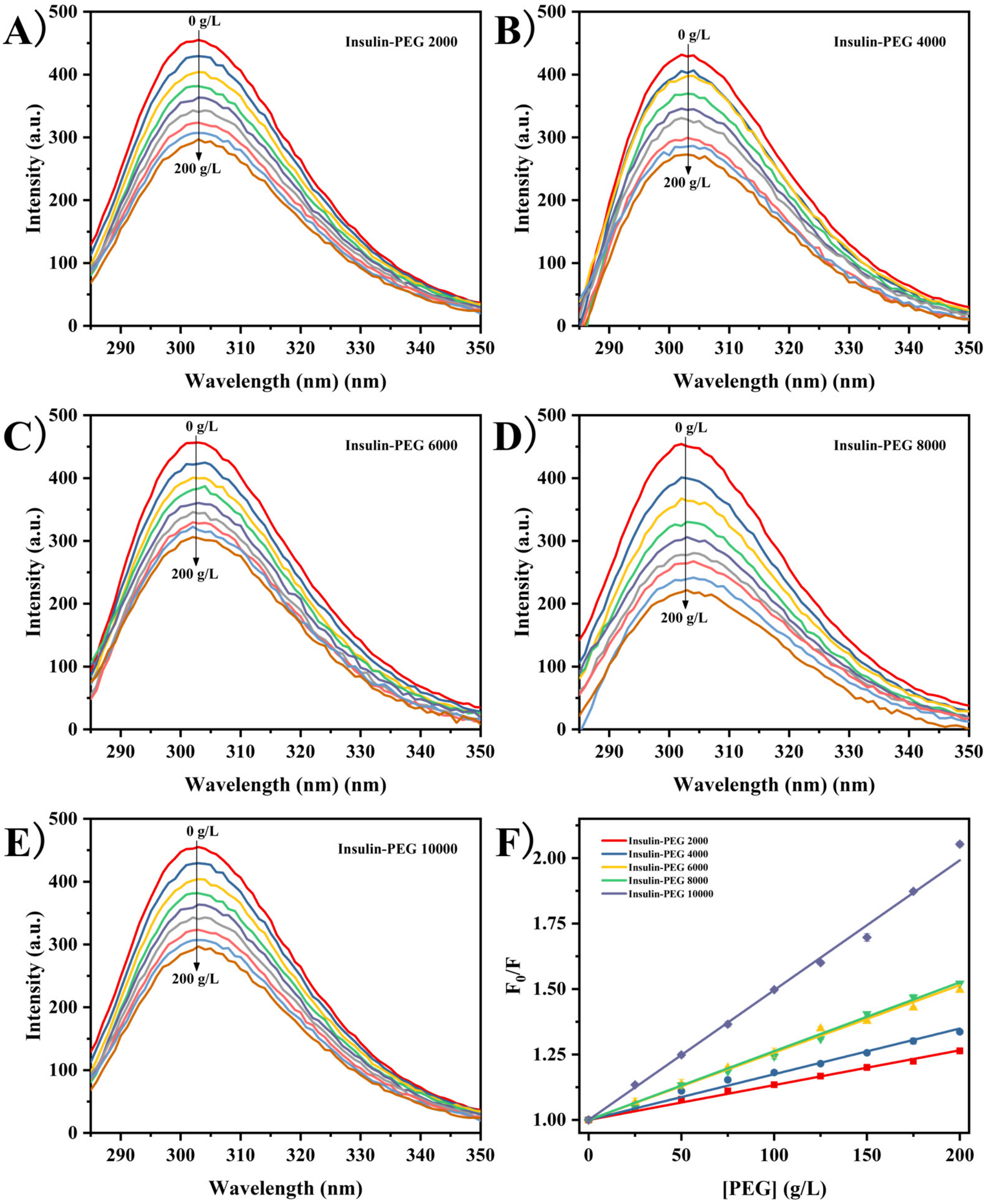

Figure S2. The effect of PEG 2000 (A), PEG 4000 (B), PEG 6000 (C), PEG 8000 (D) and PEG 10000 (E) on the fluorescence spectra of insulin. Stern-Volmer curves (F) of insulin incubated with five crowded reagents.

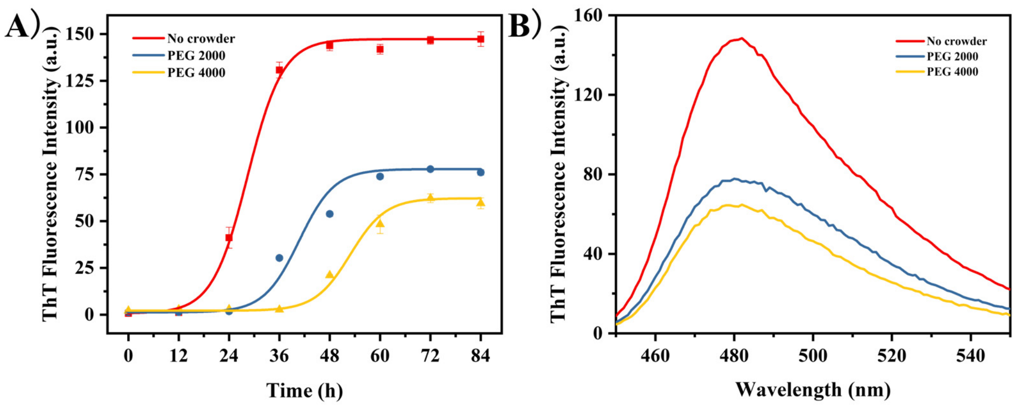

Figure S3. The aggregation kinetic curve of insulin samples without inhibitors (A) and the ThT fluorescence spectra of insulin samples after 84 h of incubation (B) in three research environments.

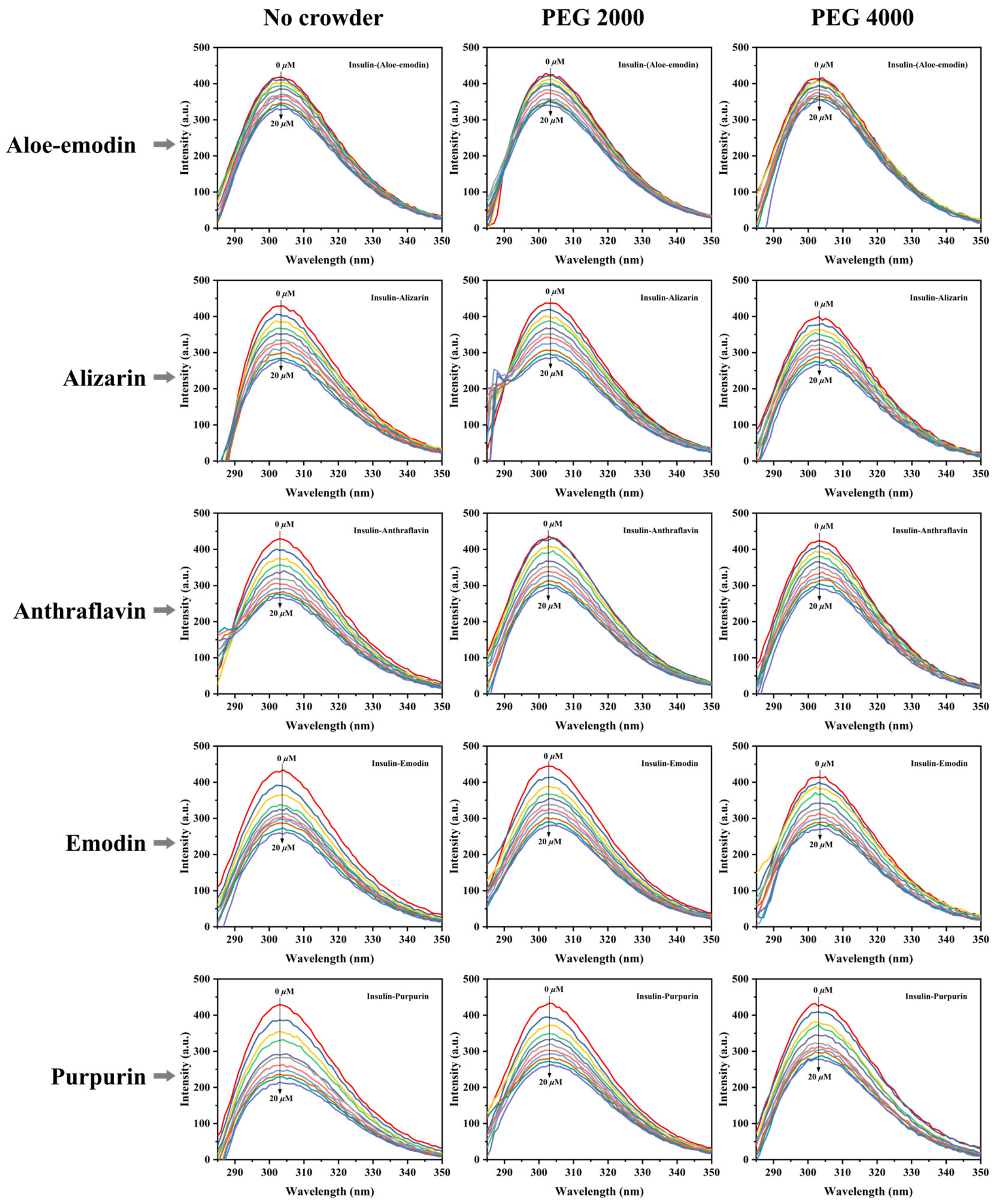

Figure S4. Fluorescence spectra of the interaction of aloe-emodin/alizarin/anthraflavin/emodin/purpurin with insulin.

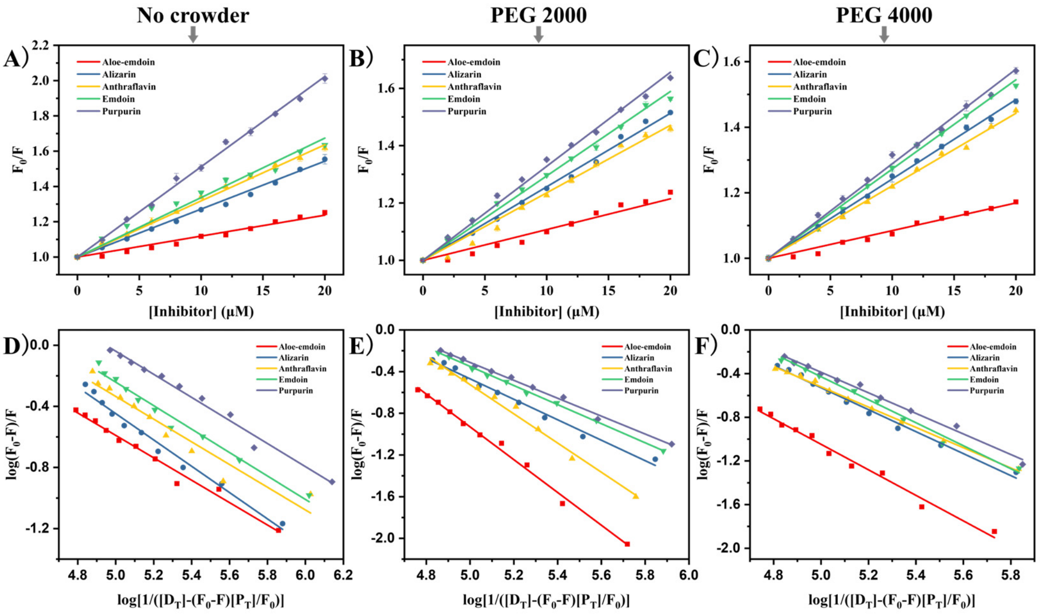

Figure S5. Stern-Volmer plots (A-C) and double-logarithmic plots (D-F) of the interaction of insulin with five anthraquinones in three research environments.

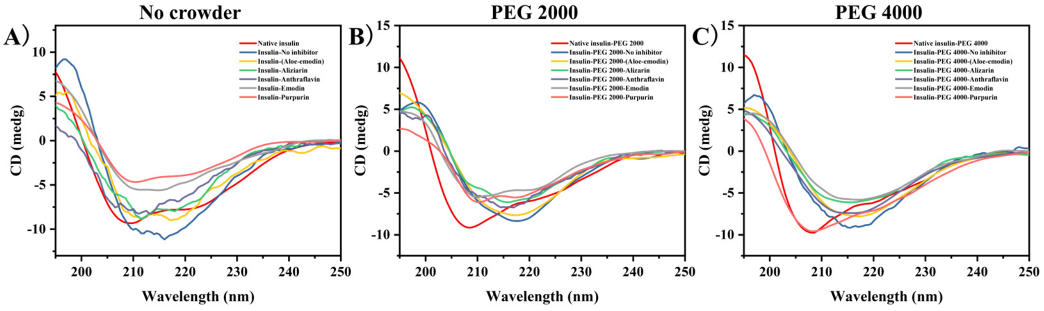

Figure S6. CD spectra of insulin incubated with five anthraquinones in dilute solution (A), PEG 2000-induced crowded environment (B) and PEG 4000-induced crowded environment (C) after 84 h of incubation.

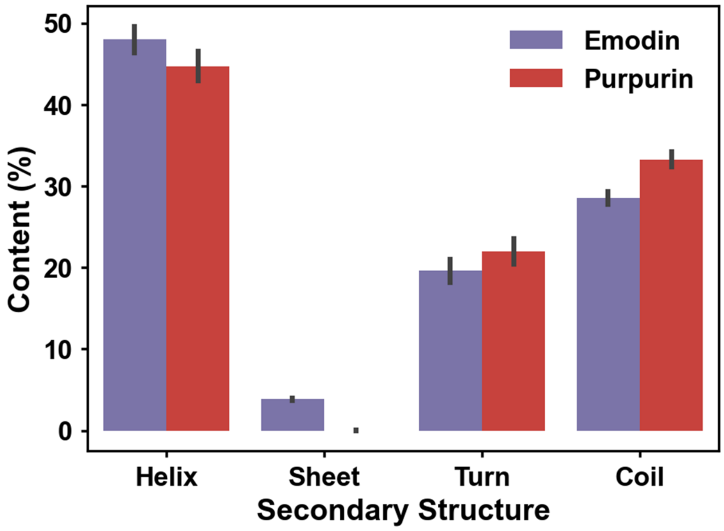

Figure S7. The secondary structure residue counts of insulin incubated with emodin and purpurin after 100 ns MD simulations.

Tables

Table S1. The Stern-Volmer quenching constant (K_{sv}) and bimolecular quenching rate constant (k_q) of the interaction between insulin and PEG 2000/ PEG 4000/PEG 6000/ PEG 8000/PEG 10000.

Systems	K_{sv} (M^{-1})	k_q (10^8 M^{-1} s^{-1})
Insulin-PEG 2000	2.66 ± 0.04	2.66 ± 0.04
Insulin-PEG 4000	7.00 ± 0.16	7.00 ± 0.16
Insulin-PEG 6000	15.42 ± 0.24	15.42 ± 0.24
Insulin-PEG 8000	21.04 ± 0.32	21.04 ± 0.32
Insulin-PEG 10000	49.60 ± 0.90	49.60 ± 0.90

Table S2 Effects of five anthraquinones on the kinetic parameters of insulin aggregation in three research environments.

Systems	Inhibitors	t_{lag} (h)	Intensity	Amyloid (%)
No crowder	No inhibitor	18.2 ± 0.4	150.4 ± 4.6	100.0
	Aloe-emodin	28.2 ± 0.1	52.4 ± 1.0	34.8
	Alizarin	27.8 ±0.2	43.9 ±2.8	29.2
	Anthraflavin	33.0 ±0.3	29.6 ±0.7	19.7
	Emodin	32.7 ± 0.3	23.1 ± 1.7	15.4
	Purpurin	32.4 ± 0.1	20.6 ± 1.7	13.7
PEG 2000	No inhibitor	31.1 ± 0.4	77.8 ± 4 .0	100.8
	Aloe-emodin	31.8 ± 0.6	31.1 ± 3 .0	40.3
	Alizarin	43.3 ±0.5	15.4 ±0.5	19.9
	Anthraflavin	34.2 ±0.0	20.5 ±0.4	26.6
	Emodin	56.3 ± 0.3	12.2 ± 0.8	15.8
	Purpurin	60.3 ± 0.4	7.6 ± 0.9	9.8
PEG 4000	No inhibitor	41.2 ± 1.0	63.5 ± 2.0	100.0
	Aloe-emodin	60.1 ± 0.9	31.5 ± 1.0	49.6
	Alizarin	64.7 ±0.1	13.3 ±0.9	21.0
	Anthraflavin	68.3 ±0.5	26.7 ±1.5	42.0
	Emodin	65.3 ± 1.2	11.3 ± 1.7	17.8
	Purpurin	80.1 ± 3.2	3.4 ± 0.9	5.4

Table S3 Secondary structural contents of insulin in the absence and presence of five anthraquinones after 84 h of incubation.

Systems	Inhibitors	α-helix	β-sheet	Others
No crowder	Native insulin	0.451	0.113	0.436
	No inhibitor	0.179	0.526	0.295
	Aloe-emodin	0.273	0.391	0.336
	Alizarin	0.359	0.225	0.416
	Anthraflavin	0.352	0.229	0.419
	Emodin	0.383	0.188	0.429
	Purpurin	0.402	0.168	0.430
PEG 2000	Native insulin	0.458	0.109	0.433
	No inhibitor	0.184	0.505	0.311
	Aloe-emodin	0.264	0.404	0.332
	Alizarin	0.331	0.254	0.415
	Anthraflavin	0.305	0.288	0.407
	Emodin	0.357	0.223	0.420
	Purpurin	0.371	0.200	0.429
PEG 4000	Native insulin	0.437	0.145	0.418
	No inhibitor	0.201	0.481	0.318
	Aloe-emodin	0.246	0.421	0.333
	Alizarin	0.306	0.298	0.396
	Anthraflavin	0.288	0.326	0.386
	Emodin	0.339	0.240	0.421
	Purpurin	0.412	0.163	0.425

Table S4 Sizes of the insulin-emodin/purpurin systems in three research environments.

Sizes (nm)	No crowder	PEG 2000	PEG 4000
No inhibitor	1196.0	953.7	819.4
Insulin-emodin system	290.4	455.1	341.5
Insulin-purpurin system	190.8	447.6	109.3

Table S5 Basic information and *in silico* lipophilicity parameters prediction.

Molecule	IUPAC name	Canonical SMILES	MW	Consensus Log P	GI absorption	Lipinski Violations
Alizarin	1,2-dihydroxyanthracene-9,10-dione	O=C1c2ccccc2C(=O)c2c1ccc(c2O)O	240.21	2.02	High	0
1,8-dihydroxyanthraquinone	1,8-dihydroxyanthracene-9,10-dione	Oc1cccc2c1C(=O)c1c(C2=O)cccc1O	240.21	2.04	High	0
Aloe-emodin	1,8-dihydroxy-3-(hydroxymethyl)anthracene-9,10-dione	OCc1cc(O)c2c(c1)C(=O)c1c(C2=O)c(O)ccc1	270.24	1.5	High	0
Chrysophanol	1,8-dihydroxy-3-methylanthracene-9,10-dione	Cc1cc(O)c2c(c1)C(=O)c1c(C2=O)c(O)ccc1	254.24	2.38	High	0
Rhein	4,5-dihydroxy-9,10-dioxo-9,10-dihydroanthracene-2-carboxylic acid	Oc1cc(cc2c1C(=O)c1c(C2=O)cccc1O)C(=O)O	284.22	1.47	High	0
Purpurin	1,2,4-trihydroxyanthracene-9,10-dione	Oc1c(O)cc(c2c1C(=O)c1ccccc1C2=O)O	256.21	1.88	High	0
1,4-dihydroxyanthraquinone	1,4-dihydroxyanthracene-9,10-dione	Oc1ccc(c2c1C(=O)c1ccccc1C2=O)O	240.21	2.15	High	0
Anthraflavin	2,6-dihydroxyanthracene-9,10-dione	Oc1ccc2c(c1)C(=O)c1c(C2=O)cc(cc1)O	240.21	1.93	High	0
Emodin	1,3,8-trihydroxy-6-methylanthracene-9,10-dione	Cc1cc(O)c2c(c1)C(=O)c1c(C2=O)c(O)cc(c1)O	270.24	1.87	High	0
Physcion	1,8-dihydroxy-3-methoxy-6-methylanthracene-9,10-dione	COc1cc(O)c2c(c1)C(=O)c1c(C2=O)c(O)cc(c1)C	284.26	2.27	High	0
